# Purinergic Signaling in Spermatogenesis

**DOI:** 10.3389/fendo.2022.867011

**Published:** 2022-04-05

**Authors:** Nadine Mundt, Lina Kenzler, Marc Spehr

**Affiliations:** ^1^ Department of Physiology, University of California, San Francisco, San Francisco, CA, United States; ^2^ Department of Chemosensation, Institute for Biology II, RWTH Aachen University, Aachen, Germany; ^3^ Research Training Group 2416 MultiSenses – MultiScales, RWTH Aachen University, Aachen, Germany

**Keywords:** spermatogenesis, ATP - adenosine triphosphate, purinoceptor, calcium signaling, P2X, P2Y

## Abstract

Adenosine triphosphate (ATP) serves as the essential source of cellular energy. Over the last two decades, however, ATP has also attracted increasing interest as an extracellular signal that activates purinergic plasma membrane receptors of the P2 family. P2 receptors are divided into two types: ATP-gated nonselective cation channels (P2X) and G protein-coupled receptors (P2Y), the latter being activated by a broad range of purine and pyrimidine nucleotides (ATP, ADP, UTP, and UDP, among others). Purinergic signaling mechanisms are involved in numerous physiological events and pathophysiological conditions. Here, we address the growing body of evidence implicating purinergic signaling in male reproductive system functions. The life-long generation of fertile male germ cells is a highly complex, yet mechanistically poorly understood process. Given the relatively sparse innervation of the testis, spermatogenesis relies on both endocrine control and multi-directional paracrine communication. Therefore, a detailed understanding of such paracrine messengers, including ATP, is crucial to gain mechanistic insight into male reproduction.⁠

## Spermatogenesis

**Figure 1:**
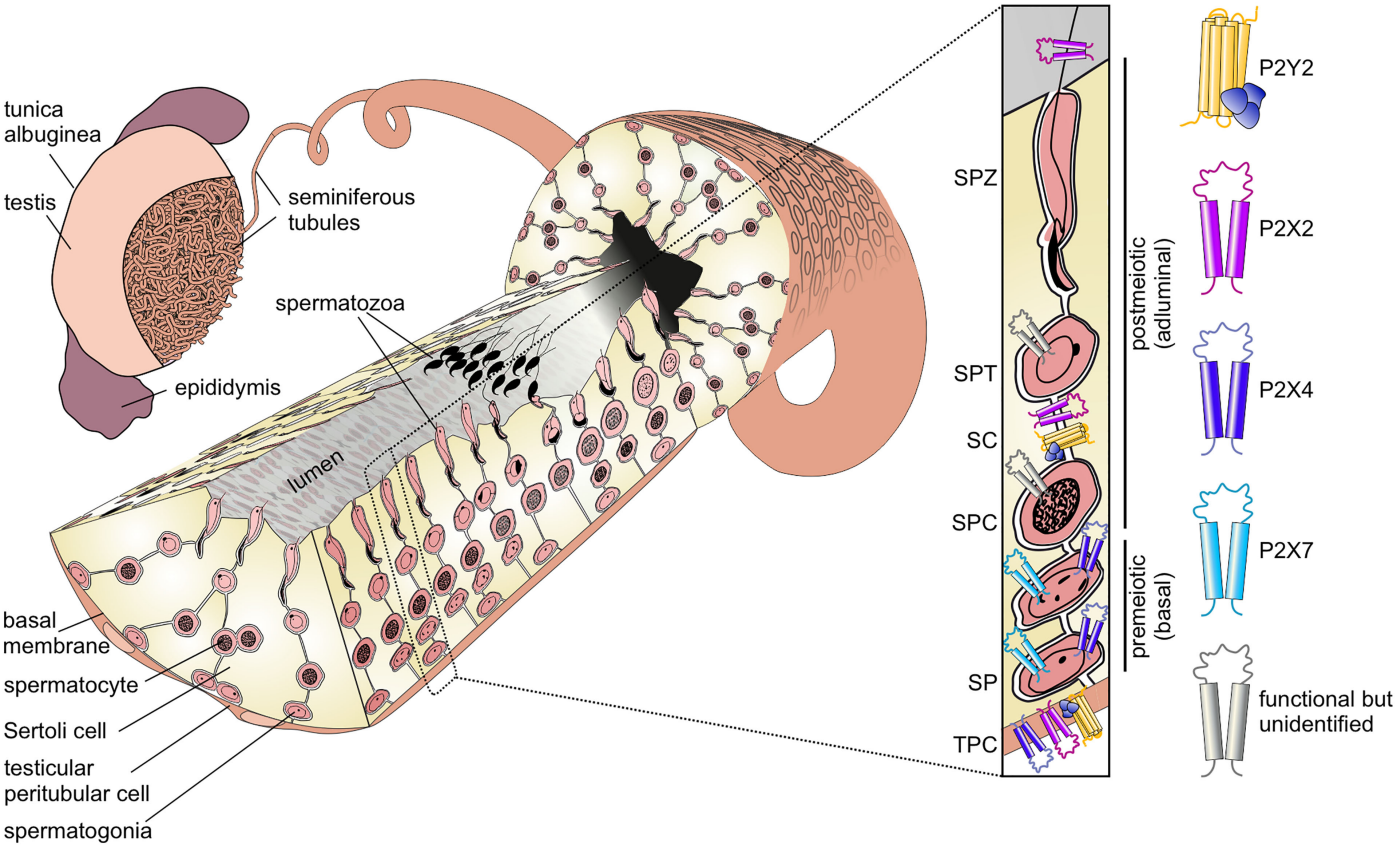
Functional P2 receptor isoform distribution among individual cell types of the seminiferous tubule. Left: Schematic illustration of the mammalian testis and cellular architecture of a seminiferous tubule. A single layer of contractile testicular peritubular cells (TPC) lines the tubular wall. Developing germ cells are distributed between nourishing Sertoli cells (SCs). Undifferentiated spermatogonia (SP) are located near the basal membrane. Spermatocytes (SPC) migrate to the adluminal compartment, where they complete meiosis. The resulting haploid round spermatids (SPT) differentiate into elongated spermatids and, eventually, into highly condensed and compartmentalized spermatozoa (SPZ). These mature, yet immotile spermatozoa are then released into the lumen (spermiation) and undergo further maturation steps once transported to the epididymis. Adapted from: Fleck, Kenzler et al. (3). Right: Distribution of P2 isoforms in various cell types of the seminiferous tubule. Schematic shows the P2 receptor distribution as supported by direct functional (i.e., physiological) evidence.

The generation of fertile spermatozoa is one of the most complex, yet least understood developmental processes in postnatal life. Spermatogenesis describes the differentiation and maturation of diploid spermatogonial stem cells into haploid spermatozoa (1). Spermatogenesis occurs in the seminiferous tubules within the mammalian testis (2) (**Figure 1**). These hollow tubules are coiled loops that converge in the *rete testis*, which feeds into the epididymis (2, 4). Seminiferous tubules comprise a specialized tissue subdivided into three compartments: the lumen, the germinal epithelium, and the tubular wall. The latter is composed of extracellular matrix proteins and flat smooth-muscle-like testicular peritubular cells (TPCs). The germinal epithelium comprises two cell types: somatic Sertoli cells and developing germ cells.

Sertoli cells fulfill essential structural, regulatory, and nourishing functions for the surrounding germ cells. They span from the basal lamina to the lumen and are associated with up to 50 germ cells (5). During the course of differentiation, Sertoli and germ cells remain connected, enabling continuous bidirectional communication. In the basal seminiferous epithelium, Sertoli cells form necklace-like tight junction threads between adjacent Sertoli cells, creating a tight barrier between the basal and adluminal compartments (6). This blood-testis barrier prevents passage of many molecules and migrating immune cells into the inner, adluminal compartment and, thus, creates a protective, immune-privileged environment for postmeiotic germ cell development (7).

During maturation, germ cells migrate in a complex process from the basal compartment towards the lumen. The first wave of spermatogenesis is initiated upon puberty and divided into four phases (8, 9):

1. Mitotic proliferation of diploid spermatogonia (spermatogoniogenesis)
2. Meiotic division of tetraploid spermatocytes into haploid spermatids
3. Morphological differentiation of spermatids into spermatozoa (spermiogenesis)
4. Sperm release into the tubular lumen (spermiation)

The first mitotic division is asymmetrical as one daughter cell remains in the stem cell pool, while the other spermatogonium is irreversibly determined to differentiate. In subsequent mitotic divisions into various spermatogonial subtypes, the cells lose contact with the basal lamina (10). Due to incomplete cytokinesis, premeiotic germ cells stay connected *via* cytoplasmic bridges allowing small molecule exchange and, hence, synchronized development (11, 12). Spermatogonia then differentiate into primary spermatocytes, which progress through meiosis and cross the blood-testis barrier. Haploid spermatids undergo drastic morphological changes (spermiogenesis), yielding elongated and flagellated spermatozoa that are located close to the tubular lumen. Finally, in a process called “spermiation”, spermatozoa are released into the lumen, which marks the endpoint of spermatogenesis (2, 8). Upon release, spermatozoa remain immotile and, thus, need to be actively transported towards *rete testis* and epididymis, where they gain the capacity for motility but remain quiescent (13–15). Sperm transport is mediated by coordinated smooth muscle contractions of TPCs that surround individual tubules (3, 16).

The bewildering complexity of cell types that coexist in the seminiferous epithelium as well as the numerous proliferation and differentiation steps that must be precisely orchestrated pose an obvious question: Which multi-directional cellular communication mechanisms control spermatogenesis?

Given the lack of pronounced seminiferous tubule innervation testicular sympathetic innervation appears restricted to blood vessels and the tunica albuginea (17), spermatogenesis relies on endo-, auto-, and paracrine communication pathways. Therefore, a detailed understanding of the relevant paracrine messengers, including ATP, promises to provide much needed mechanistic insight into male reproduction. ⁠

## Purinergic Signaling

One of the paracrine messengers that has attracted increasing scientific interest in a multitude of general physiological events is extracellular adenosine triphosphate (ATP) (18–21). Through an evolutionarily conserved route for cell-to-cell communication, extracellular ATP activates members of the membrane-bound P2 purinoceptor family (18). ATP-gated P2 receptors are divided into two classes, namely ionotropic P2X receptors (22, 23), and metabotropic P2Y receptors, which are members of the G protein-coupled receptor (GPCR) superfamily (24). The majority of the eight P2Y receptor isoforms (P2Y1, 2, 4, 6, 11) couple to G**_α_**_q_, thus signaling *via* phosphoinositide turnover. G**_α_**_q_ activates phospholipase C, which in turn hydrolyzes phosphatidylinositol-4,5-bisphosphate to inositol-1,4,5-trisphosphate (IP_3_) and diacylglycerol. Cytosolic increase in IP_3_ level triggers Ca^2+^ release from internal Ca^2+^ storage organelles (i.e., the endo/sarcoplasmic reticulum) *via* IP_3_ receptors. The main effector of P2Y12, P2Y13, and P2Y14 is G_αi/o_ followed by an activation or inactivation of adenylate cyclase and altered cytosolic cyclic adenosine monophosphate (cAMP) levels (25).

P2X receptors, by contrast, are homo- or heterotrimeric ligand-gated nonselective cation channels. They share a common transmembrane topology – intracellular termini and two transmembrane domains separated by a large extracellular loop (26) – with DEG/ENaC/ASIC channels. Upon ATP binding, conformational changes lead to the opening of a cation-permeable channel pore (27). Among the P2X family, seven homotrimeric (P2X1–7) and several heterotrimeric isoforms have been described, all of which share substantial Ca^2+^ permeability, but are readily distinguished by ligand affinities, activation and desensitization kinetics, as well as distinct pharmacological fingerprints (28). The complexity of both receptor families, which cover a vast dose-response range of effective ATP concentrations, and the broad spatiotemporal response scales of P2 receptors confer both functional specificity and physiological flexibility to a ubiquitous signaling pathway. Accordingly, a given cell’s P2 receptor expression profile underlies its unique response phenotype upon ATP exposure. Notably, as both metabotropic and ionotropic ATP response pathways represent substantial cellular Ca^2+^ gates, purinoceptors mediate numerous Ca^2+^-dependent downstream effects, including control of gene transcription, protein phosphorylation, ion channel function, muscle contraction, and more (29). While the general picture is still incomplete, we here seek to summarize evidence from a growing number of reports about purinergic signaling routes within the seminiferous tubule and their potential implications in spermatogenesis and male (in)fertility.

### Purinoceptor Signaling in Germ Cells

Given the broad physiological response scale of purinoceptors, purinergic signaling has been proposed to play a role in controlling germ cell maturation at different developmental stages. In mice, twelve such stages are sequentially transitioned to complete one seminiferous epithelial cycle. Accordingly, immunohistochemical investigation of cell type- and stage-dependent protein expression has been notoriously difficult. Early work described immunoreactivity for several P2X receptor subtypes in the rat testis (30). Various germ cell types throughout different stages of the seminiferous epithelial cycle were found immunopositive for P2X2, P2X3, and P2X5 receptors. By contrast, P2X4 and P2X6 receptors appeared absent from rat testis samples – a finding that was later contradicted by Ko and coworkers (31). P2X1 receptors were exclusively detected in blood vessels and P2X7 antibody staining was restricted to Sertoli cells (30). Notably, P2X2 and P2X3 isoforms, which frequently form functional heteromers in the nervous system (32), were usually observed in the same cell types and stages (30).

**Figure 2:**
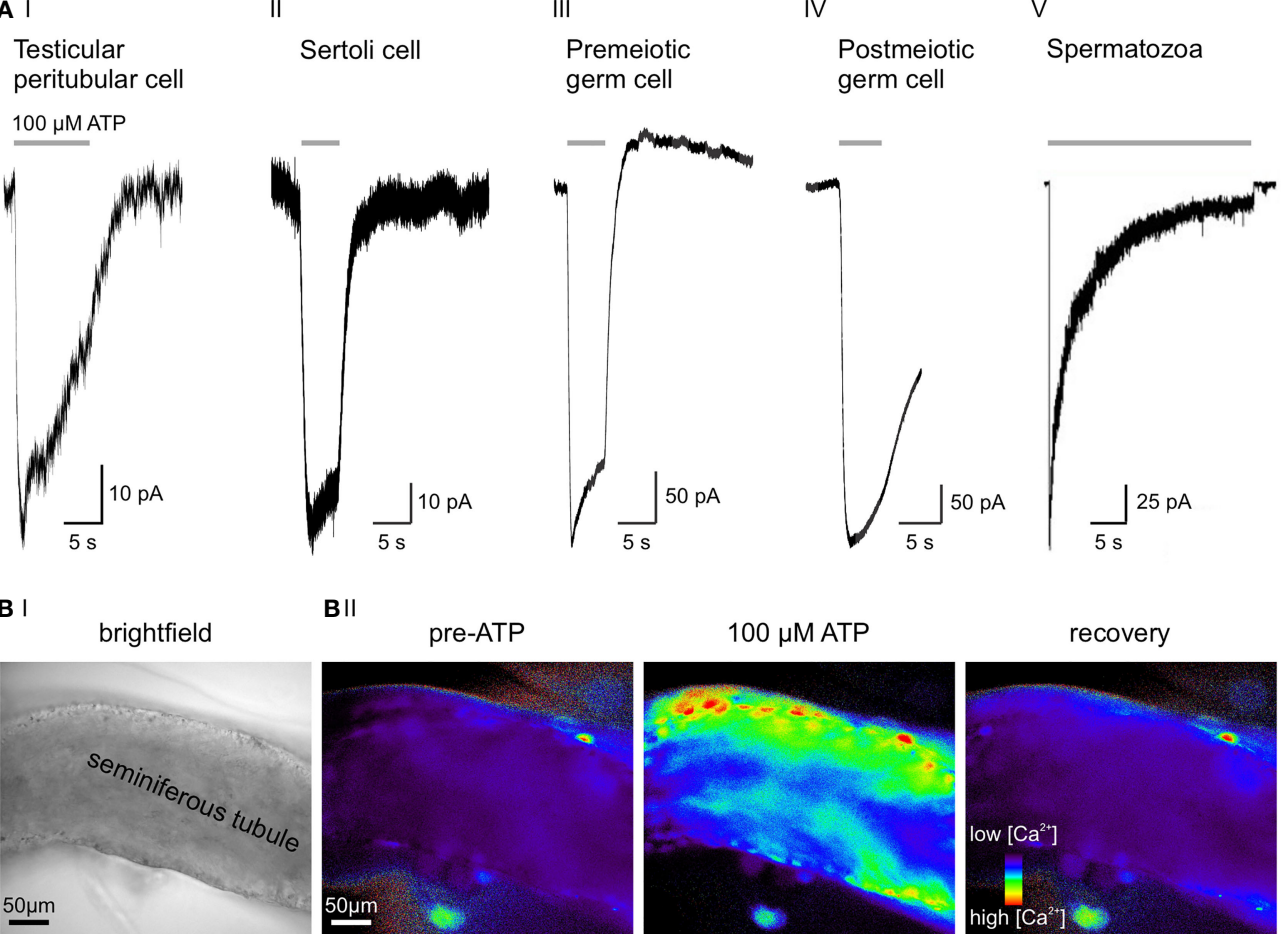
ATP sensitivity across cell types of the seminiferous tubule. **(A)** Representative whole-cell voltage-clamp recordings from various testicular cell types, transiently exposed to extracellular ATP (100 µM). Negative current indicates cation influx through P2X receptors. **(A_I_)** Slowly desensitizing P2X2 and/or P2X4 current in a mouse TPC (3). **(A_II_)** ATP activates P2X2 in murine Sertoli cells (33). **(A_III_)** 100 µM ATP selectively activates P2X4, but not P2X7 in premeiotic spermatogonia. Note the delayed BK-mediated outward current (33). **(A_IV_)** Postmeiotic germ cells exhibit an ATP-induced inward current, but the underlying P2X isoform is yet to be identified (unpublished data; recording in an acute seminiferous tubule section from an adult mouse according to (33), extracellular solution containing (mM) 145 NaCl, 5 KCl, 1 CaCl^2^, 0.5 MgCl^2^, and 10 HEPES; pH = 7.3, intracellular solution containing (mM) 143 KCl, 2 KOH, 1 EGTA, 0.3 CaCl^2^, 10 HEPES, and 1 Na-GTP ([Ca^2+^]_free_ = 110 nM); pH = 7.1, stimulation with 100 μM ATP for 5 s). **(A_V_)** Epididymal mouse spermatozoa with characteristic fast-activating and slowly desensitizing P2X2 current evoked by extracellular ATP. Electrophysiological recording was performed on a head plus midpiece fragment by Navarro et al. (2011) (34). **(B)** Combined ionotropic and metabotropic ATP responses of various cells in an acute seminiferous tubule section visualized as Ca^2+^-dependent changes in fluorescence. Imaging was performed according to published experimental protocols (35). **(B_I_)** Brightfield micrograph of the seminiferous tubule section under investigation. **(B_II_)** Fluorescence images of the same seminiferous tubule bulk-loaded with fura-2/AM (30 μM, 30 min at room temperature). Pseudocolor images (rainbow 256 color map) illustrate relative cytosolic Ca^2+^ concentration before, during, and after ATP stimulation (unpublished data)⁠.

Recently, we combined gene expression analysis, immuno- and bioanalytical chemistry, protein knockdown, and single-cell electrophysiology to gather functional evidence for purinergic signaling in male germ cells (33). We identified a multidimensional ATP response pathway consisting of both P2X4 and P2X7 receptors and downstream Ca^2+^-activated large conductance (BK) K^+^ channels in prepubescent mouse spermatogonia (**Figure 2A_III_**). P2X4 and P2X7 receptors display distinct ATP affinities, and their activation triggers transmembrane currents with characteristic kinetics that enable unequivocal electrophysiological isoform identification. Cooperatively activated by concurrent membrane depolarization and increased cytoplasmic Ca^2+^, hyperpolarizing BK channels provide a negative feedback mechanism that counteracts the effects of P2X receptor activation and ensures swift repolarization of the spermatogonial membrane potential (33).

While some of the apparent discrepancies between the above studies (30, 31, 33) likely result from species [mouse (33) *versus* rat (30, 31)] and/or age [juvenile (33) *versus* adult (30, 31)] differences, they also highlight the limitations of unidirectional (i.e., immunochemistry-only) protein expression analysis. Electrophysiological recordings from postmeiotic germ cells in acute seminiferous tubule slices of adult mice are technically challenging. Our own unpublished data nonetheless indicate functional expression of a fast activating and slowly desensitizing ATP-activated channel in postmeiotic spermatocytes and/or round spermatids (**Figure 2A_IV_**). The molecular identity of this putative P2X receptor remains to be identified.

Given the emerging role of extracellular ATP in numerous physiological signaling processes, it is tempting to speculate that spermatozoa might be exposed to varying concentrations of extracellular ATP in the testis, epididymis, and/or female reproductive tract. ATP might, therefore, play a role in modulating sperm fertilizing capacity. In humans, extracellular ATP has been reported to increase the fertilizing potential of sperm and, accordingly, sperm exposure to ATP during IVF treatment has been suggested (36). Early studies report that extracellular ATP triggers acrosome exocytosis in human sperm *via* P2X-dependent Na^+^ influx (37, 38). In rat spermatozoa, P2X7 has been proposed to mediate the ATP-triggered acrosome reaction (39). While the acrosomal membrane is as yet inaccessible to electrophysiological recordings, acrosomal P2X receptor currents remain to be verified. A different mechanism was found in bovine spermatozoa, where extracellular ATP appears to activate P2Y receptors. The resulting elevation in cytoplasmic Ca^2+^ activates PKCα, which triggers acrosomal exocytosis (40). In 2007, Edwards et al. quantified the effects of extracellular ATP on acrosomal exocytosis, protein tyrosine phosphorylation, and sperm motility parameters in human sperm (41). In healthy and asthenozoospermic donors, ATP had no impact on acrosome exocytosis or tyrosine phosphorylation. However, it significantly altered sperm motility, increasing curvilinear velocity and percentage of hyperactivation. This observation might explain the previously described benefits of ATP supplement during IVF treatment.

Navarro et al. reported a nonselective cation current in the midpiece of mouse spermatozoa that is activated by external ATP exposure (**Figure 2A_V_**) (34). This current matches the kinetics and pharmacological profile reported for recombinant P2X2 and, importantly, is absent in P2X2^-/-^ mice. Despite the loss of this ATP-gated current, P2X2^-/-^ spermatozoa show unaltered motility and acrosome reaction. However, P2X2^-/-^ males are subfertile when given the chance to mate at high frequencies, indicating that P2X2 adds a selective advantage under frequent mating conditions. The authors hypothesize that increased intracellular Ca^2+^ through P2X2 energizes sperm mitochondria in the midpiece, presumably as a consequence of Ca^2+^-dependent potentiation of enzymes in the Kreb’s cycle (42).

### Purinoceptor Signaling in Sertoli Cells

Work from multiple laboratories suggests that extracellular ATP triggers a rapid and transient increase in the cytosolic Ca^2+^ concentration of Sertoli cells, albeit with partly conflicting propositions for the underlying purinoceptor isoforms (31, 33, 43–47).

Endocrine control of spermatogenesis along the hypothalamic–pituitary–testicular axis converges on Sertoli cells (48). Sertoli cell function is centrally regulated by gonadotropins, either directly by follicle stimulating hormone (FSH) or indirectly by luteinizing hormone-dependent generation of dihydrotestosterone. FSH surges trigger cAMP production and mobilization of cytosolic Ca^2+^ in Sertoli cells (48). Interestingly, both ATP and its uridine derivative UTP inhibit FSH-dependent cAMP accumulation by 70% in rat Sertoli cells, suggesting that P2Y2 or P2Y4 receptors are involved (43). Moreover, rapid IP_3_ accumulation was observed upon ATP exposure in primary cultures of rat and mouse Sertoli cells, in line with P2Y2 or P2Y4 receptor activation (49). In rat Sertoli cells, extracellular ATP evoked 17β-estradiol production/secretion. This effect depended on both membrane depolarization *via* Na^+^ influx (implicating P2X receptors) and Ca^2+^ release from internal stores (suggesting a concurrent role of P2Y receptors) (47).

Both receptor identification and direct functional characterization of purinergic signaling in mouse Sertoli cells were performed by Veitinger et al. in 2011 as well as Fleck et al. in 2016 (33, 45). P2X2 and P2Y2 are the prevailing purinoceptors (**Figure 2A_II_**) with confirmatory results obtained from both Sertoli cell–germ cell co-cultures (45) and acute seminiferous tubule sections (33). These (electro-)physiological observations are in accordance with early findings by Foresta et al. in rat Sertoli cells. Here, the authors claimed that ATP exposure generates both an increase in cytosolic Ca^2+^ by release from intracellular stores (P2Y receptors) and a depolarizing Na^+^ influx consistent with P2X receptor activation (43). Notably, Veitinger and coworkers establish that mitochondria serve as essential regulatory components of Sertoli cell purinergic Ca^2+^ signaling (45).

### Purinoceptor Signaling in Testicular Peritubular Cells

Spermatogenesis completes with the release of still immotile spermatozoa from the seminiferous epithelium into the lumen of the seminiferous tubule. After detachment from Sertoli cells, sperm must therefore be transported towards the *rete testis* and epididymis for further maturation. Accordingly, precisely regulated tubular transport mechanisms are imperative for reproduction.

Early on, observations of minute motions of seminiferous tubule segments (50, 51) have sparked speculation about a critical role for smooth muscle-like TPCs (52, 53) in male (in)fertility through mediating contractile tubule movements (54, 55). However, direct experimental *in vivo* evidence for paracrine control of TPC contractions has been lacking (56) and quantitative live-cell measurements of seminiferous tubule contractions are rare and controversial (57–60). Somewhat surprisingly, early work explicitly excluded extracellular ATP as an activator of TPCs (61). By contrast, we recently reported both ATP-dependent Ca^2+^ signals and adenosine-dependent proinflammatory actions in human TPCs *in vitro* (62, 63). Notably, we also identified purinergic signaling pathways as physiological triggers of tubular contractions both *in vitro* and *in vivo*. By acting on ionotropic (P2X2 and/or P2X4) and metabotropic (P2Y2) purinoceptors (**Figure 2**), extracellular ATP elevates cytosolic Ca^2+^ (**Figure 2B**), activates TPC contractions, and triggers stage-dependent directional sperm movement within the mouse seminiferous tubules (3). Combining recordings from primary mouse and human TPC cultures as well as acute mouse seminiferous tubule slices with intravital multiphoton imaging of intact tubules, we provide direct and quantitative evidence for purinergic TPC signaling that triggers robust peristaltic movement of luminal sperm (3). Electrophysiological and Ca^2+^ imaging data suggest that, while metabotropic P2Y signaling is sufficient to induce ATP-dependent contractions, influx of extracellular Ca^2+^ through ionotropic P2X receptors enhances TPC contractions. While the full picture is admittedly still incomplete, current data support a concept of Ca^2+^-induced Ca^2+^ release mechanisms that amplify ATP-dependent excitation-contraction coupling.

Being under androgen control, expression of TPC contractility proteins initiates with puberty and, notably, TPC-selective androgen receptor knock-out renders mice infertile (64). Both findings underscore a potential role of TPC contractions in male (in)fertility. Accordingly, pharmacological targeting of purinergic signaling pathways to (re)gain control of TPC contractility represents an attractive approach for male infertility treatment or contraceptive development (3). Still, translation of TPC contractions and their putative role(s) from mice to humans awaits further physiological investigation.

## Concluding Remarks

With recent technical advances in male reproductive physiology, we and others identified functional P2X and/or P2Y receptors in essentially all cell types of the seminiferous tubule, constituting a purinergic signaling network (**Figure 2**). Local ATP elevations will affect the surrounding cells within a limited paracrine radius both electrophysiologically and biochemically by triggering membrane depolarization as well as substantial Ca^2+^ influx and cAMP signaling. Distinct type- and stage-specific purinoceptor repertoires will determine unique response profiles of individual target cells. Moreover, ectonucleotidases provide pathways of local ATP degradation/metabolization, restricting the effective range of paracrine ATP signaling (65). Both Sertoli and germ cells have been proposed as putative ATP release sites (66), but a conclusive picture of extracellular ATP release in the testis requires future investigation.

## Author Contributions

NM and LK acquired the data that is indicated as unpublished. MS contributed to the conceptualization thereof. NM wrote the first draft of the manuscript and designed the figures. LK and MS wrote sections of the manuscript. All authors contributed to the article and approved the submitted version.

## Funding

This work has been funded by the Deutsche Forschungsgemeinschaft (DFG), grant reference number 368482240/GRK2416 (NM and MS).

## Conflict of Interest

The authors declare that the research was conducted in the absence of any commercial or financial relationships that could be construed as a potential conflict of interest.

## Publisher’s Note

All claims expressed in this article are solely those of the authors and do not necessarily represent those of their affiliated organizations, or those of the publisher, the editors and the reviewers. Any product that may be evaluated in this article, or claim that may be made by its manufacturer, is not guaranteed or endorsed by the publisher.
